# Revolving hexameric ATPases as asymmetric motors to translocate double-stranded DNA genome along one strand

**DOI:** 10.1016/j.isci.2023.106922

**Published:** 2023-05-19

**Authors:** Margaret Bohmer, Abhjeet S. Bhullar, Tao Weitao, Long Zhang, Jing-Huei Lee, Peixuan Guo

**Affiliations:** 1 Center for RNA Nanobiotechnology and Nanomedicine, The Ohio State University, Columbus, OH, USA; 2 College of Pharmacy, Division of Pharmaceutics and Pharmacology, The Ohio State University, Columbus, OH, USA; 3 College of Medicine, Dorothy M. Davis Heart and Lung Research Institute and James Comprehensive Cancer Center, The Ohio State University, Columbus, OH, USA; 4 Interdisciplinary Biophysics Graduate Program, College of Art and Science, The Ohio State University, Columbus, OH 43210, USA; 5 Center for the Genetics of Host Defense UT Southwestern Medical Center, Dallas, TX, USA; 6 Department of Biomedical Engineering, College of Engineering and Applied Science, University of Cincinnati, Cincinnati, OH, USA

**Keywords:** Biochemistry, Molecular biology, Molecular Structure

## Abstract

DsDNA translocation through nanoscale pores is generally accomplished by ATPase biomotors. The discovery of the revolving dsDNA translocation mechanism, as opposed to rotation, in bacteriophage phi29 elucidated how ATPase motors move dsDNA. Revolution-driven, hexameric dsDNA motors have been reported in herpesvirus, bacterial FtsK, *Streptomyces* TraB, and T7 phage. This review explores the common relationship between their structure and mechanisms. Commonalities include moving along the 5′→3′ strand, inchworm sequential action leading to an asymmetrical structure, channel chirality, channel size, and 3-step channel gating for controlling motion direction. The revolving mechanism and contact with one of the dsDNA strands addresses the historic controversy of dsDNA packaging using nicked, gapped, hybrid, or chemically modified DNA. These controversies surrounding dsDNA packaging activity using modified materials can be answered by whether the modification was introduced into the 3′→5′ or 5′→3′ strand. Perspectives concerning solutions to the controversy of motor structure and stoichiometry are also discussed.

## Graphical abstract

**Figure:**
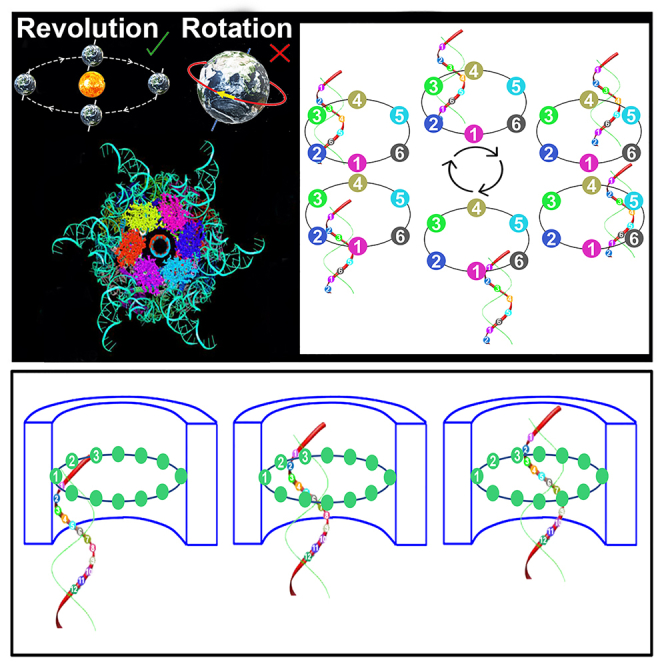

## Introduction

One of the basic features of life is motion, which is realized by the ubiquitous biomotors that belong to the ATPase family. A fundamental question is how the living system moves its lengthy helical, double-stranded DNA (dsDNA) genome without coiling and tangling. If translocation of a dsDNA genome uses a rotational mechanism, the resulting lengthy dsDNA genome will be supercoiled or intertwined. Thus, dsDNA translocation has been of interest for many years.

The simple structure of bacteriophage DNA packaging motors has provided intriguing models for the study of dsDNA translocation. Bacteriophages rely on DNA-packaging motors during replication to translocate their DNA genome into pre-formed protein shells known as procapsids. These DNA-packaging motors are among the strongest molecular machines found in nature, capable of generating over 50 pN of force,^1^ as compared to the actomyosin motor which can generate about 6 pN of force.^2^ It is well-accepted that these motors convert chemical energy from ATP hydrolysis into mechanical energy.^3^ However, the exact mechanisms as to how this is achieved remain blurred.

**Figure 1:**
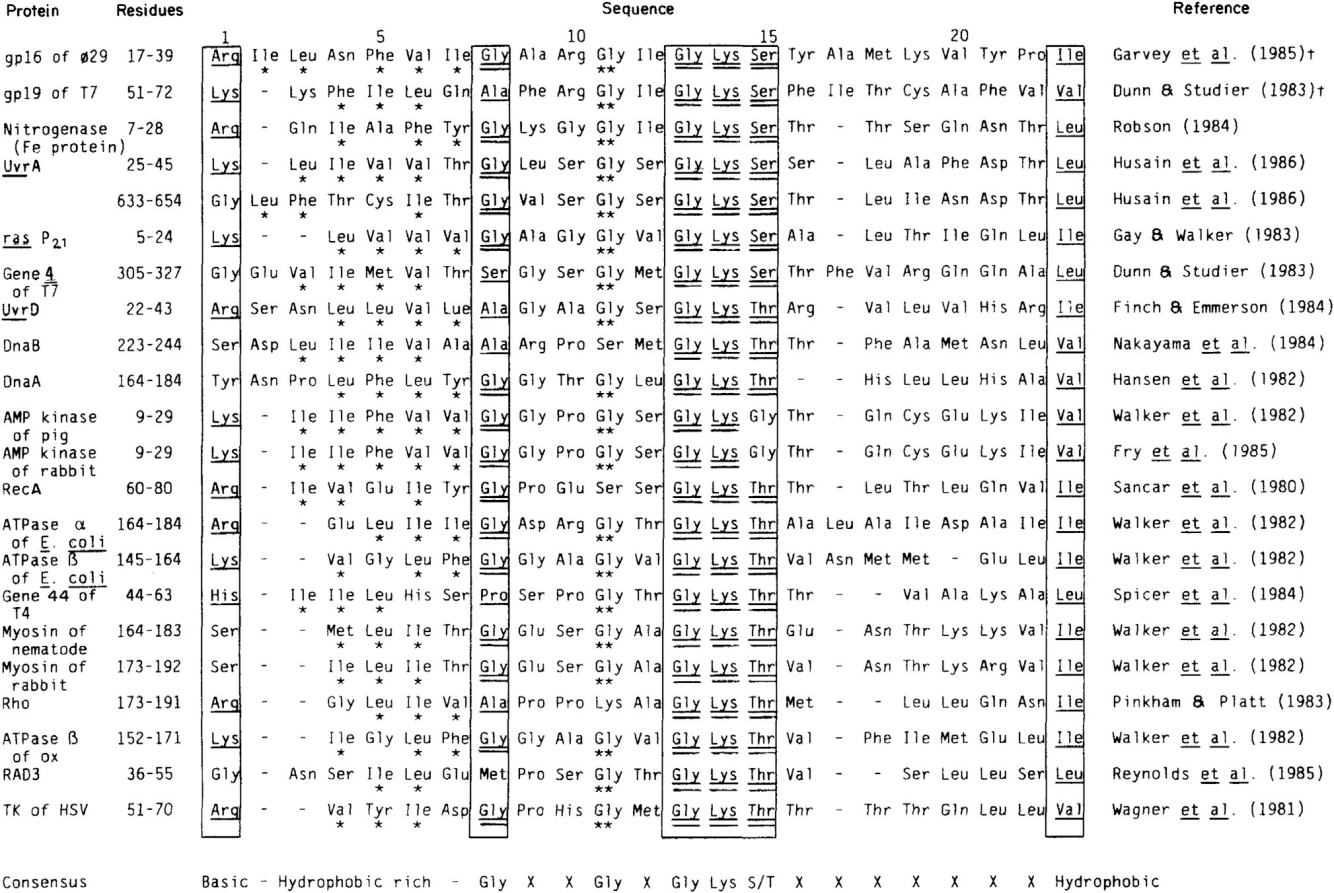
The first report to reveal that DNA translocation motors are driven by an ATPase Strong sequence homology (as is boxed) details that many DNA-translocating proteins contain ATP-binding motifs.^3^ Identical amino acids are double-underlined and chemically similar amino acids are single-underlined. ∗ Refers to hydrophobic residues. ∗∗ refers to conserved glycine residues.

As both sequencing and structural biology techniques advanced, similarities concerning the mechanism of DNA translocation began to emerge. The ATPase proteins share some commonly conserved motifs^3^ (Figure 1), such as the Walker A and Walker B motifs, that are involved in ATP binding and hydrolysis.^4^^,^^5^^,^^6^ In addition, these biomotors contain hydrophobic regions between the ATP-binding region and conserved glycine and lysine sequences next to a serine or threonine residue within the ATP-binding consensus sequence. It is interesting to note that sequence homology hinted almost 40 years ago that many different biomotors interact with DNA and ATP in similar ways^3^ (Figure 1), yet single-molecule and structural techniques have advanced enough only recently for these similarities to be seen in action.^7^^,^^8^^,^^9^^,^^10^

This review summarizes how the components of bacteriophage, bacterial, and eukaryotic virus biomotors assemble and communicate. We also describe the structural and mechanistic similarities among these motors that utilize the common revolution mechanism. These similarities of revolving ATPases include the channel wall charity, motor component stoichiometry, channel size related to dsDNA, revolution along one strand, sequential inchworm style, asymmetry, and electrostatic configurations. The challenges of structural investigation into the motors, as well as potential solutions, are also discussed.

### The phi29 packaging motor uses a revolving mechanism without rotation

Phi29, a dsDNA bacteriophage that infects *Bacillus subtilis,* is an attractive and well-studied system for revealing how biomotors move lengthy dsDNA strands. The components of the biomotor can be readily produced^11^ and it functions with great efficiency during *in vitro* studies.^12^ Part of the phi29 biomotor is a funnel-shape tunnel that connects the viral capsid to the tail. This connector is composed of a dodecameric ring of gp10 protein that connects it to the viral capsid,^13^^,^^14^ viral packaging RNA (pRNA),^15^^,^^16^^,^^17^ and gp16 protein, which acts as an ATPase.^3^^,^^18^

Initially, it was proposed that bacteriophage DNA-translocation motors moved DNA through a rotating “nut and bolt” mechanism, in which the portal acted as a nut and the DNA acted as the bolt that was propelled into the procapsid by rotational force.^13^^,^^19^ This rotary mechanism was later challenged by the fact that the T4 phage’s biomotor could still pack DNA when it was immobilized.^20^ Further studies using fluorescence polarization illustrated that there was no rotational movement of the phi29 biomotor during DNA transport.^21^ In addition, the diameters of the biomotor pores for phi29 and T4 are ∼3.6 nm, making them much larger than dsDNA (∼2 nm) and therefore unlikely to utilize rotation.^22^,^23^ Furthermore, T4 and phi29 biomotors display left-handed chirality, opposite to the right-handed chirality of dsDNA.^24^ Single-channel DNA translocation conductance assays demonstrated that neither phi29 nor T4 use a rotational mechanism but rather a revolving one in which the DNA strand revolves around the inner wall of the pore.^24^ Both phi29^18^ and T4^25^ rely on conformational changes within the ATPase ring to drive dsDNA into a viral capsid.

Data from cryogenic electron microscopy (cryoEM) showed that dsDNA formed a unique toroid structure around the phi29 connector’s N-terminal channel during entry.^26^ X-ray crystallography showed that protein subunits of the channel wall were tilted 30° with respect to the central axle.^27^^,^^28^^,^^29^ CryoEM imaging also revealed that the T7 phage’s dsDNA did not go through the center of the connector channel but moved along the wall.^30^^,^^31^ Evidence from functional analyses ruled out a rotational mechanism,^20^^,^^22^ and single-molecule fluorescence experiments showed no 360° full rotation of the DNA within the biomotor channel but rather a 1.5° rotation per base pair translocated, which is caused by conformational changes of the connector^24^^,^^32^

**Figure 2:**
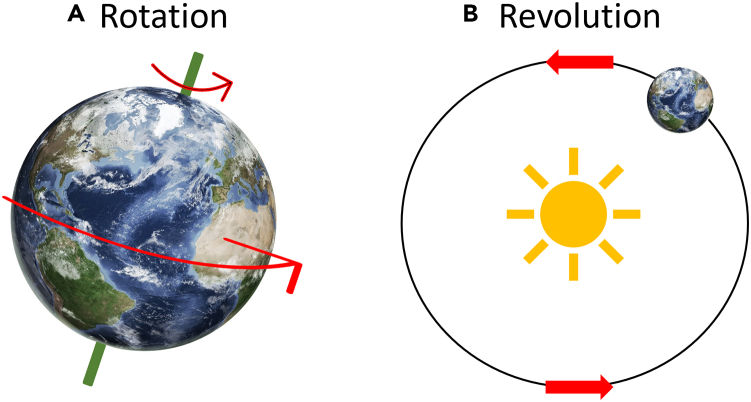
Rotating versus Revolving Mechanism in DNA Translocation (A) Rotation is an object turning around its axis, such as the Earth completing one rotation every 24 h. Rotating biomotors have pore diameters similar in size to the diameter of the substrate, ensuring a tight fit. (B) Revolution is the orbital motion of an object around another object, such as the Earth making a revolution around the sun every 365 days. Biomotors utilizing the revolution mechanism contain pore diameters larger than that of the dsDNA.

Consequently, it has been reported that the phi29 DNA-packaging motor moves dsDNA by revolution, without rotation, of the DNA.^28^^,^^33^^,^^34^ An example of rotation is the Earth spinning on its own axis (Figure 2A). Instead, the DNA moves around the wall of the biomotor channel in a revolving motion, analogous to the Earth’s revolution around the sun (Figure 2B). This discovery of a new type of DNA translocation resolved the 35-year mystery of how packaging motors move DNA into viral capsids.^28^^,^^33^ The structural similarities of DNA-translocating channels have prompted researchers to delve further into how each of the channel components work together to transport DNA. One common structural theme is ATPase proteins arranged in a ring, with conformational changes in these proteins influencing their affinity for DNA.^18^^,^^35^^,^^36^^,^^37^ Many biomotors utilizing the revolution mechanism also contain an arginine finger between the ATPase proteins.^34^^,^^38^^,^^39^^,^^40^ The arginine finger has been found to be a starter of the phi29 biomotor,^40^ and additional studies have shown how the finger interacts with the ATPase subunits.^34^^,^^41^ In general, there are several key steps to the revolution mechanism: (1) motor assembly at the entrance to the capsid, (2) ATP binding, (3) conformation changes of the biomotor proteins that set the stage for (4) DNA binding, (5) ATP hydrolysis, and (6) DNA advancement.^42^

### Other dsDNA translocation motors contain revolving ATPases

Many other DNA translocases exhibit similar structures to phi29 and use the same revolving mechanism. DNA translocases utilizing the revolving mechanism display hexameric rings of the ATPase subunits with a central channel wider than that of dsDNA. The T7 DNA-ejection motor,^7^ herpesvirus DNA-packaging motor,^7^^,^^8^ a bacterial translocase,^43^ and a bacterial plasmid-conjugating enzyme^44^ are examples of hexameric ATPases that revolve dsDNA.

#### T7 bacteriophage hexameric ejectosome for dsDNA ejection during infection

**Figure 3:**
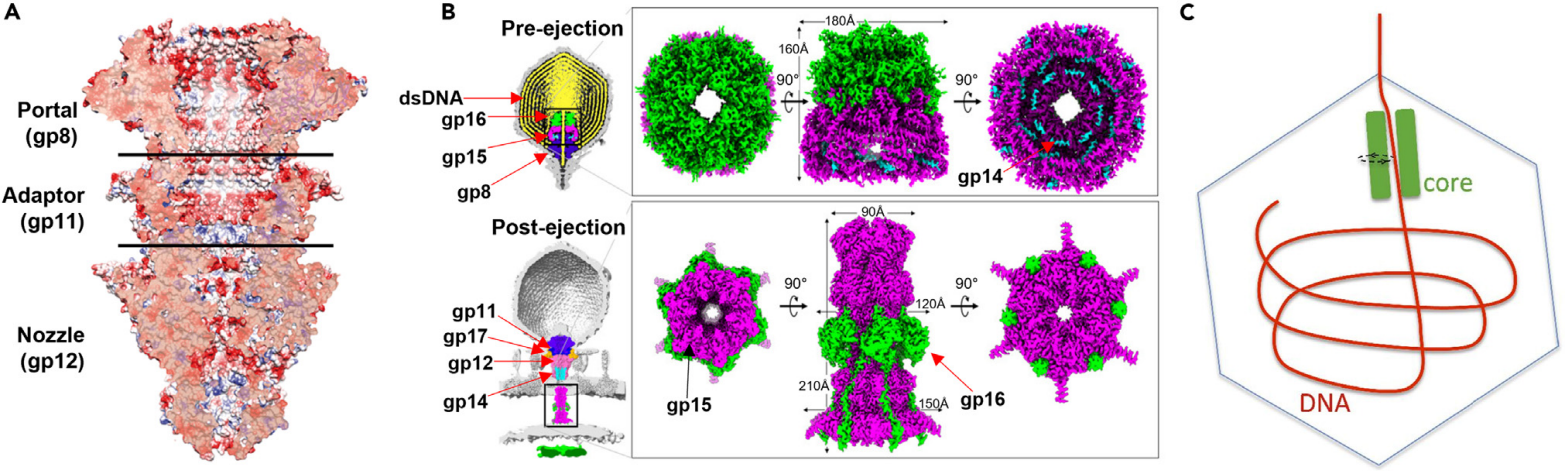
T7 DNA-packaging and ejection machines (A) Electrostatic potential of the T7 tail (blue = positive, red = negative, white = neutral) (reproduced with permission from Nature).^7^ (B) CryoEM maps of the T7 ejectosome pre- and post-dsDNA ejection. The ejectosome (gp14, gp15, gp16) descends to create a channel for the DNA to travel through. Components include the genome (yellow), tail complex (gp11 in dark blue and gp12 in light pink), portal protein (gp8 in purple), and ejectosome (gp14 in cyan, gp15 in magenta, and gp16 in green).^49^ (C) Diagram of DNA being ejected circularly, but not from the center of the channel, indicating revolution.^30^

Tailed dsDNA phages rely on their tails to adsorb onto and inject their genomes into the host cell. For the T7 phage, this tail contains four proteins: a connector (gp8), tubular proteins (gp11 and gp12), and fibers (gp17). After the phage DNA is packaged into the prohead, the ATPase (terminase) detaches and is replaced by gp11, the adaptor protein. Gp11 binds to the gp8 connector, forming a dodecameric, toroidal adaptor.^7^ This gp8-gp11 complex is mostly electronegative, possibly to avoid adherence of the dsDNA-complex to reduce the force and energy required during DNA packaging and ejection^7^^,^^45^^,^^46^ (Figure 3A). After gp11 attaches to gp8, gp12 then assembles at the bottom of the tail to build a hexameric nozzle. Six gp17 fibers then attach to the connection point between gp11 and gp12, making a complete tail.^47^^,^^48^ The gp12 is organized around a large, central β-propeller domain, and each protomer is left-hand twisted.^7^ Many electrostatic interactions exist between gp11 and gp12. The part of gp12 that attaches to gp11 is highly electropositive, whereas gp11 is highly electronegative^7^ (Figure 3A).

The portal exists in two conformations: open or closed.^7^ During T7 DNA packaging, the terminase stabilizes the open conformation, allowing the DNA to pass into the procapsid.^7^ Conversely, when DNA packaging is finished and the terminase detaches, the closed conformation is favored, preventing DNA from sliding out. The portal conformation reverts to open when gp11 binds to gp8. When this happens, the DNA travels down the tail channel where the gp12 nozzle prevents leakage.^46^^,^^47^ It has been proposed that this mechanism of β-propeller loops closing DNA ejection channels might exist in other phages, such as P22, in which its gp10 tail protein connects its adaptor and tail needle (gp26).^7^^,^^50^ When the T7 phage is ready to infect a host cell, it releases gp14, gp15, and gp16 proteins, which stack on top of one another to form concentric rings.^30^ These proteins form the DNA-ejectosome, a channel through which viral DNA is injected through the membrane into the host cell^49^^,^^51^ (Figure 3B). Through this channel, gp15 and gp16 eject DNA in a circular fashion^30^ (Figure 3C), supporting a revolving mechanism.^33^^,^^35^

#### Herpesvirus DNA-packaging motor

The herpesvirus DNA-translocating motor contains a hexameric ring and each subunit in the ring is made up of three parts: pUL28 and pUL33, which help regulate DNA translocation, and pUL15, which acts as an ATPase and a nuclease.^8^ Working together, pUL33, pUL28, and pUL15 push DNA through a dodecameric portal into the viral capsid. CryoEM studies performed on the herpesvirus motor in the absence of ATP and in the presence of a non-hydrolyzable ATP analog (to lock the biomotor in its “active” state) point toward a DNA translocation mechanism that resembles that of phi29.^8^ The internal channel of the herpesvirus biomotor is 3.9 nm, making it much wider than the diameter of dsDNA (∼2 nm) and similar to the larger diameters of the phi29 and T4 bacteriophage motor channels.^52^ Furthermore, the pUL15 hexamer relies on conformational changes induced by ATP hydrolysis to move DNA through the channel; each subunit in the pUL15 hexamer works together by one subunit inserting an arginine finger into the adjacent subunit to induce ATP hydrolysis.^8^ When the arginine finger was mutated, pUL15 was still able to form a hexamer but did not exhibit ATPase activity.^8^ By studying the conformational changes occurring from ATP hydrolysis, including the movement of the arginine finger from one subunit to the adjacent one, the authors concluded that this biomotor transports DNA in a sequential, revolving manner through the motor channel.^8^^,^^53^

#### FtsK bacterial DNA translocase

Similar mechanisms for DNA translocation have been seen in bacteria. An example of a bacterial dsDNA translocase is Ftsk, which is found in many bacterial species, including *Pseudomonas aeruginosa* and *Escherichia coli.*^54^ Like the phi29 and T4 biomotors, the FtsK DNA translocation motor is made up of three parts: α, β, and γ. The α and β domains create a hexamer that hydrolyzes ATP whereas the γ domain is responsible for binding to specific DNA sequences.^54^ Recent cryoEM studies done on the FtsK motor in the process of transporting DNA have shown how conformational changes around the hexameric ring drive DNA movement,^43^ the same theme seen in phi29, T4, and the herpesvirus biomotors.

The data suggest that each catalytic step induces a conformational change in one subunit, prompting concerted conformational changes in all the other subunits.^43^ During these conformational changes, the angle of the β domain changes relative to the α domain. These changes allow dsDNA to be passed from subunit to subunit as it moves through the motor channel.^43^ As DNA passes through the channel, it interacts with basic amino acid residues along the channel wall that form a spiral staircase, which correlates exactly with the grooves in the DNA double helix.^43^ The αβ subunit at the top of the spiral staircase adopts a conformation that lowers its affinity for DNA and releases ADP, whereas the subunit at the bottom of the staircase binds to DNA and ATP. As the ATP is hydrolyzed, it drives conformational changes for all subunits at once. The subunits each move through a series of 6 conformations as the DNA revolves around the ring, wherein each catalytic cycle adds an ATP-bound subunit at the bottom of the spiral staircase and removes an ADP-bound subunit from the top in a treadmilling fashion.^43^ Hexameric rings using ATP hydrolysis to drive conformational changes that transport DNA, which is regulated by an arginine finger, appears to be a common theme among DNA translocases.^34^^,^^40^

Furthermore, SpoIIIE, a gram-positive bacterial DNA translocase, and the mimivirus’ dsDNA-packaging machine have been predicted to also use a revolving mechanism. SpoIIIE is in the same protein family as Ftsk and the two proteins share many structural similarities.^55^ In adidtion, there is evidence that, during DNA translocation, SpoIIIE only contacts the 5’→3′ DNA strand,^56^ which supports the revolution mechanism (see evidence of motor-dsDNA interaction with one strand supports the revolving motion mechanism). Likewise, the dsDNA translocation motor of the *Acanthamoeba polyphaga* mimivirus (APMV) is also structurally similar to FtsK, and it has been hypothesized that it, too, translocates DNA along one strand.^57^

#### TraB plasmid-conjugating protein

An additional DNA transportation motor is the bacterial TraB DNA translocase, found in *Streptomyces*, which transfers DNA from a donor to a recipient cell during conjugative genome transfer. It is also dependent on ATP hydrolysis to drive DNA movement. Negative stain electron microscopy indicated that the ATPase component of the TraB biomotor forms hexamers.^44^ Based on the hexameric structure and sequence similarity with the γ region of FtsK, it was hypothesized that TraB translocated DNA through a similar revolving mechanism. Further structural studies are still needed to characterize the conformational changes driving DNA movement. Both the T7 and TraB channels have diameters of ∼3.0 nm, larger than that of dsDNA, which hints that these motors may utilize a revolving mechanism.^43^

### Evidence of motor-dsDNA interaction with one strand supports the revolving motion mechanism

The mechanism of revolving dsDNA during packaging involves the dsDNA moving with only one strand touching the channel.^31^^,^^33^ This phenomenon is imperative to the revolving mechanism; if the dsDNA was rotating, both strands would contact the channel during translocation. The 5′→3′ motion along one strand was illuminated in 2014.^24^^,^^27^ Although the revolving mechanism was not proposed or discussed before 2014,^31^^,^^33^ much evidence in the literature substantiated the revolving mechanism, as described below. The one-strand moving phenomenon was further corroborated in 2021.^58^

As reported, DNA packaging involves the ATPase electrostatically interacting with negatively charged dsDNA.^14^^,^^18^^,^^35^^,^^36^^,^^37^ In studies on SpoIIIE, chemical modification of the dsDNA’s 3′→5′ strand to change the charge of its backbone did not affect the DNA packaging.^56^ However, alteration of the 5′→3′ strand did seriously affect DNA packaging,^56^ indicating that SpoIIIE only electrostatically interacts with the backbone of the 5′→3′ strand. These results support the finding of the revolution mechanism reported by the Guo group,^31^^,^^33^ showing that only one strand of the dsDNA interacts with the motor channel during revolution.

**Figure 4:**
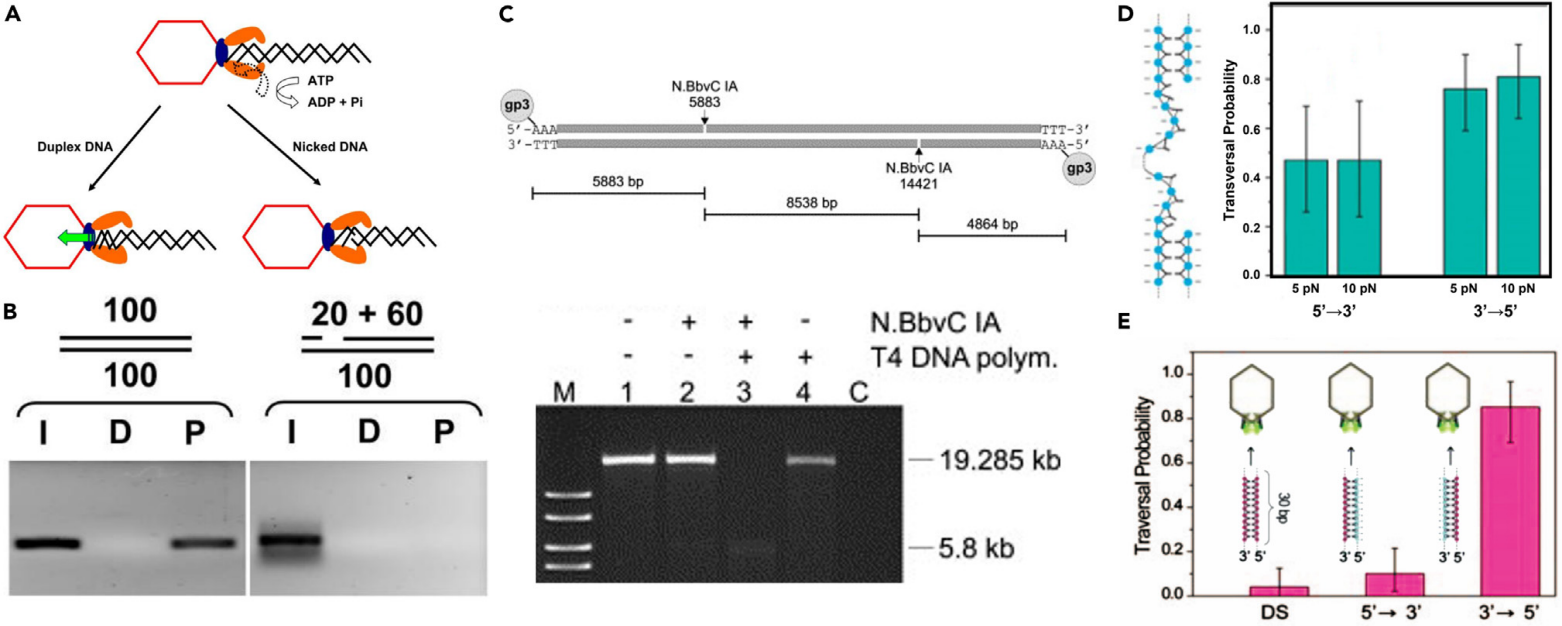
DsDNA translocation of revolving motors moves only around one strand, not two strands, of the dsDNA (A) Diagram illustrating how T4 cannot package nicked DNA (prohead = red; gp17 terminase = orange, gp20 portal = blue, DNA = black.^61^ (B) Differences in T4 DNA packaging between normal dsDNA (left) and DNA with a 20-bp gap (right). I= dsDNA positive control, D= No ATP negative control, P= packaging experimental.^61^ (C) Demonstration of phi29 dsDNA-gp3 packaging blockage by single-stranded gaps created by N.BbvC IA in the presence of the T4-DNA polymerase. Only the left-end fragment (before the gap) of phi29 genomic DNA is packaged.^59^ (D) Blockage of phi29 DNA packaging by 20-nt ssDNA gaps on the 5’→3′ strand but not the 3’→5′ strand.^63^ (E) DNA packaging is blocked by chemical modification of the negatively charged phosphate backbone on one strand. Blue is normal dsDNA, and pink is methylphosphonate DNA (MeP), which is uncharged. Modification on the 3′ → 5′ strand does not block dsDNA packaging, but modification on the 5’→3′ strand seriously affects DNA packaging (reproduced with permission from Nature).^63^

The revolving mechanism and motor action along one strand of the dsDNA addresses the historic controversy of early reports on dsDNA packaging. When phi29 DNA was nicked (removal of phosphodiester bonds between nucleotides), the phi29 motor was still able to package it.^59^ This contrasts with studies on the T4 motor, which found that it was unable to translocate nicked DNA^60^^,^^61^ (Figure 4A), unless the nick was repaired with ligase.^62^ On the contrary, a later report found that only nicks in short T4 DNA substrates (100 base pairs) inhibited packaging, but nicks in long substrates (500 base pairs) were tolerated.^61^ T4 DNA-packaging assays revealed that single-stranded extensions of fewer than 12 base pairs at the DNA’s 5′ end did not inhibit translocation, whereas larger ssDNA extensions did significantly affect the packaging.^61^ In addition, they uncovered that T4 could not package DNA with a 20-base pair gap^61^ (Figure 4B). Similarly, the T4 motor is also unable to translocate a DNA-RNA hybrid.^61^

Whether or not the phi29 motor can package gapped DNA is debated, as there are conflicting reports. One study by Moll et al. found that phi29 could not package gapped DNA^59^ (Figure 4C), whereas a different study by Aathavan et al. found that gaps were tolerated.^63^ In the former study, Moll et al. used N.BbvC IA to introduce site-specific nicks into the DNA, which were then expanded into gaps with T4 DNA polymerase.^59^ However, in the latter study, Aathavan et al. concluded that gaps on the 3’→5′ strand resulted in higher packaging probability than those on the 5’→3′ strand^63^ (Figure 4D). The same study also added portions of uncharged DNA to the strands. Likewise, they found that DNA backbone modifications on the 5’→3′ strand hindered packaging, but modifications on the 3’→5′ strand did not^63^ (Figure 4E).

The controversies surrounding packaging of modified materials can be answered by the revolving mechanism. It is hypothesized that if a gap is introduced to the 3′→5′ strand, no inhibition on DNA packaging may be found.^63^ Conversely, if the gap is introduced to the 5′→3′ strand, DNA packaging would be inhibited. The walking of the motor along one dsDNA strand also explains how the T4 motor can remove intercalating dyes during translocation, suggesting that the shape of the helix changes as it moves through the T4 motor channel.^64^ Furthermore, this motion along one strand in the revolving mechanism also explains the chirality (see the 30° chirality of the connector channel supports the revolving motion mechanism) and the one-way traffic mechanism found in the phi29 DNA-packaging motor.^65^

### An inchworm sequential transition style supports the revolving motion mechanism without rotation

#### Refinement of gp16 domains in the phi29 motor

**Figure 5:**
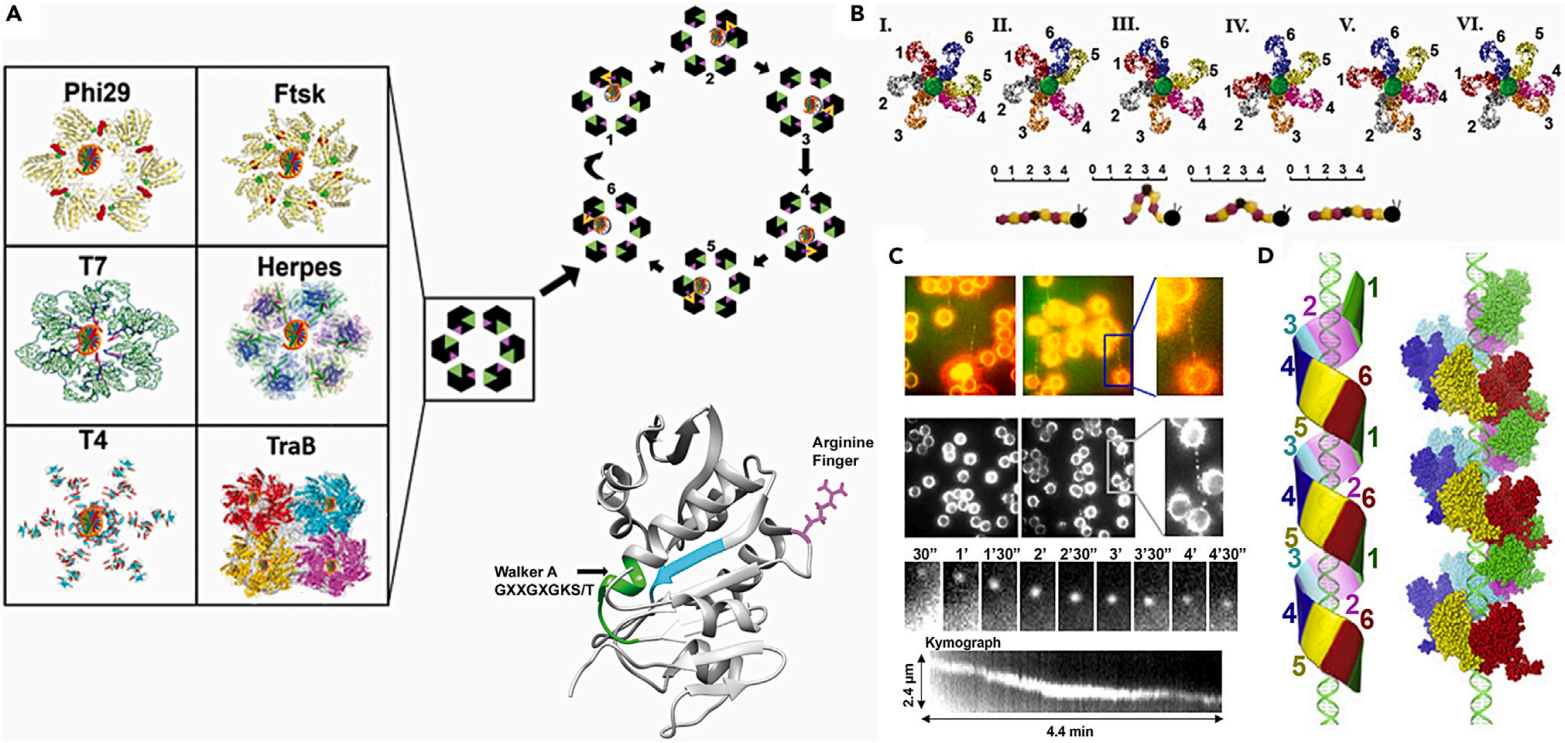
Revolving hexameric dsDNA translocation motors (A) (Left) The dsDNA translocating motors of Phi29,^38^*E. coli* Ftsk^38^ (reproduced with permission from ACS), *Streptomyces* TraB,^44^ Herpesvirus,^8^ and the contraction tail motor for dsDNA ejection of T7^68^ and T4 (PDB:3J2N)^69^; (Right) The ATPase subunit Arg finger and ATP-binding Walker domain are in pink and green, respectively. Yellow indicates dimerization. The arginine finger sticks out, as shown by the detailed crystal structure of the gp16 ATP-binding domain. (B) During dsDNA packaging, phi29 ATPase gp16s move asynchronously via sequential action, akin to how an inchworm moves. (C) Total internal reflection fluorescence microscopy showing the gp16 complex queued and moving along the dsDNA crossing the polylysine beads with (i,ii,iv) and without (iii) dsDNA tethered, characterized via kymograph.^28^ (D) A proposed model showing gp16 assembled into an open washer with six subunits queued into a left-handed spiral conformation.^27^

In the sequential inchworm model of phi29’s gp16, the C-terminal domain first anchors itself to pRNA by contacting the CCA bulge of the pRNA.^66^^,^^67^ Then, its N-terminal domain moves away from the C-terminal domain as the gp16 switches between its “compacted” and “extended” modes. This movement drives DNA through the phi29 biomotor channel into the viral capsid^8^^,^^38^^,^^44^^,^^68^^,^^69^ (Figure 5A). The interaction between the C-terminal domain and pRNA allows the N-terminal domain to propel DNA to the next gp16 subunit.^36^ This action of the ATPase’s C- and N-terminal domains coming together and moving apart drives DNA movement, like an inchworm moving by bringing its tail close to its head and then extending its body (Figure 5B).^40^^,^^70^^,^^71^ This ATP-driven movement distorts the ATPase ring, resulting in an asymmetrical hexameric structure. Such ring distortion appears to be common to both phi29^72^ and T4.^73^

#### The inchworm mechanism was first modeled by mathematical quantification

The inchworm sequential action mechanism of the phi29 DNA packaging motor was proposed based on quantification using the sensitive *in vitro* phi29 assembly system.^11^ The report found that each of the six pRNA molecules worked individually, but in a sequential style.^70^ Probability computation between a combination of wild-type and mutant pRNAs agreed with competitive inhibition results, favoring the proposal that the six pRNAs functioned via an inchworm sequential mechanism.^70^ This sequential inchworm manner explains why pRNA is so sensitive to mutation; when any one of the six pRNAs is replaced with an inactive one, the inchworm stops and DNA packaging is completely blocked.^70^ This phenomenon is analogous to a series electrical circuit, which requires that all parts are working to function. If one part of the circuit is not working, the current will not flow. Similarly, all six pRNAs should be functional for DNA to be packaged. Individual pRNAs, together with the packaging ATPase, take turns mediating successive inchworm packaging steps. Moreover, it was also reported that the arginine finger regulates the sequential inchworm mechanism.^34^^,^^40^

#### The inchworm mechanism is supported by motor conformational changes

The molecular detail of the inchworm sequential mechanism is supported by recent cryoEM data on the phi29 biomotor stalled at different points during DNA translocation. It was found that the motor can adopt different conformations, presumably to change how its components contact DNA to move it through the channel.^72^ In the ATP-bound state, the N-terminal domain of the gp16 ATPase switches to a helical conformation, allowing it to tightly bind to DNA. When all ATPase subunits are bound to ATP, the ring adopts a helical conformation with each subunit on a slightly different plane from the adjacent subunit. As the first subunit in the ring hydrolyzes ATP, its affinity for DNA lowers and DNA is released. Meanwhile, this first subunit undergoing a conformational change forces the other subunits in the ring to move and brings the adjacent subunit into the same plane as the first one. This movement translocates DNA toward the procapsid. When two adjacent subunits are in a closed position, it provides an opportunity for the *trans*-acting arginine finger from one subunit to induce ATP hydrolysis in the next subunit, continuing the cycle.^34^^,^^40^ In the absence of ATP, the gp16 subunits sit in a planar configuration. This planar conformation is perturbed into a helical conformation as ATP binds and is hydrolyzed into ADP.^72^ As the ADP molecules are released and ATP molecules bind to the gp16 ring, the N-terminal domain adopts a planar ring structure.^72^ However, it is important to note that this conformational inchworm transition between subunits, resulting in an asymmetrical hexamer, was interpreted by the researchers Woodson et al. as a pentamer.^72^ As the phi29 gp16 ring’s conformation transitions, DNA is pushed through the biomotor channel.^72^^,^^74^

The “inchworm” term has been used by others to describe a different phenomenon in the phi29 motor.^58^^,^^74^ They proposed a pentameric motor in which one of the subunits was inactive. In this model, translocation occurs in a repeating series of four “burst” steps followed by a “dwell” phase.^75^ This proposed model might not be common to other biological systems and will not be discussed in detail in this review (see Zhao et al., 2013).^32^^,^^33^^,^^76^ The reported four-step bursting and pausing may also be explained by the presence of four lysine rings within the phi29 connector (see electrostatic interaction of four electropositive lysine layers with the electronegative DNA backbone facilitates advancement along one strand and supports the revolution mechanism).

#### The inchworm mechanism is supported by molecular dynamics simulations

Molecular dynamics simulations^36^ and biochemical assays^41^ provide the basis for the modified sequential inchworm model. In the presence of pRNA, molecular dynamics simulations showed a distance of 40–60 Å between gp16’s N- and C-termini in the phi29 motor. This interaction with pRNA may anchor its C-terminal domain. A linker between the two domains provides enough flexibility for the C-terminal domain to orient itself for optimal binding to pRNA.^36^ During this pRNA binding, basic residues of gp16 may align themselves to the interior rings^77^ of the biomotor channel, facilitating electrostatic interaction between gp16 and DNA. Moreover, the interfaces between gp16 subunits in these simulations^36^ align with previous biochemical assays^34^^,^^41^ that show the arginine finger interface between adjacent gp16 N-terminal domains.^34^^,^^40^

Further molecular modeling studies were performed based on the crystal structure of the ATPase ring of the asccφ28 bacteriophage.^78^ Asccφ28 is a phi29-like phage, hence making its ATPase motor homologous to the gp16 motor of phi29. Molecular dynamics simulations were performed on this ATPase to determine the flexibility of each ATPase in the ATP-bound, ADP-bound, and apo (unbound) states with no nucleotide bound. In the apo state, the ATPase is highly flexible. When ATP is bound, the ATPase loses flexibility in its N-terminal gate and lid subdomains. “Lid” refers to the regions of the monomer that protrude perpendicularly from the channel whereas the gate is tangential to the packaging ring subunits. The lid subdomains are responsible for mediating interaction between adjacent ATPases in the ring.^78^ When ATP binds to the ATPase, the N-terminal gate and lid subdomain become less flexible. As the lid subdomain loses flexibility, it rotates toward the active site of the ATPase and draws it closer to the adjacent ATPase subunit in the ring. This ATP binding leads to a more rigid structure for dsDNA binding. When the ATP is hydrolyzed and ADP is bound to the ATPase, the lid subdomain remains less flexible, but the N-terminal gate increases in flexibility. The ATP binding and ADP unbinding cause another conformational change in the lid subdomain, driving movement of the ATPase ring and transporting DNA through it.^78^

This finding aligns with the inchworm sequential model. When the Walker A domain of a subunit of the hexameric ATPase is bound to ATP, it has high affinity for dsDNA. The ATP-bound subunit also forms a dimer with the adjacent subunit via the arginine finger.^18^^,^^34^^,^^40^ When the ATP is hydrolyzed to ADP by the Walker B domain, the subunit adopts a new conformation with low affinity for dsDNA, thus releasing the dsDNA. When released, the dsDNA is pushed to the adjacent subunit, which has ATP bound, and the cycle repeats.

#### How does the ATPase interact with DNA via an inchworm mechanism?

The existence of conformational changes in the motor raises the question as to whether DNA itself can alter its interactions with the motor by changing its conformation. Fluorescence studies on the T4 biomotor indicated that DNA “crunched” itself from the B form to the A form as it moved through the motor channel.^64^^,^^79^^,^^80^ Although the DNA conformational switch was a passive result of its passage through the motor channel, it could be part of the integrated action of the inchworm process. This became the basis for the “scrunch worm” model, in which DNA transitions from the B form to the A form as a passive physical phenomenon during DNA translocation.^75^^,^^81^

Additional studies on phi29’s gp16 provide more clues to how each gp16 subunit interacts with DNA. To further elucidate what happens at the gp16 ring during DNA translocation, the X-ray crystallographic structure of gp16’s C-terminal domain^36^ was compared with previous structures of analogous components of the T4 motor^82^ and of phi29’s gp16 N-terminal domain in molecular dynamics simulations. Structurally, gp16’s C-terminal domain resembles the C-terminal domain of T4’s gp17 in that they both contain RNase H-like nuclease domains.^36^^,^^82^ Despite their similar structures, the protein sequences are different. This can account for the fact that C-terminal domains of DNA packaging ATPases, including T4’s gp17, possess nuclease activity but the C-terminal domain of phi29’s gp16 does not.^36^^,^^82^

Even though gp16 does not exhibit any nuclease activity, it can still bind to nucleic acids. This RNase H-like nuclease domain may provide a way for phi29’s gp16 to interact with pRNA and/or DNA.^36^ Such dual interaction may play a role in the coordinated inchworm action of pRNA and gp16 to translocate DNA. Molecular dynamics simulations and comparison with other DNA-binding structures indicate that the C-terminal domain of phi29’s gp16 can theoretically bind to DNA in two different orientations: a vertical one, as seen in human RNase H1^83^; and a horizontal one, as seen in the *Thermus thermophilus* RuvC/Holiday junction complex.^36^^,^^84^ CryoEM studies indicated that there are some contacts between the C-terminal domain and DNA, and these contacts more closely resemble the vertical interaction.^72^ Nuclear magnetic resonance (NMR) studies also showed direct contact between the DNA and C-terminal domain.^85^ In addition, a surface site containing many positively charged residues among a flexible region provides a site for transient DNA binding,^36^ aiding the ability of one gp16 subunit to pass the DNA strand to the next gp16 subunit in the ATPase ring.^34^^,^^40^ As ATP is hydrolyzed, an arginine finger from one gp16 subunit interacts with the gp16 adjacent to it to form a non-covalent dimer that brings the two subunits closer to one another.^34^ An arginine finger mutation in one gp16 subunit completely abolishes the gp16 biomotor ring’s ability to translocate DNA,^34^^,^^40^^,^^76^ supporting other structural and biochemical analyses indicating a high degree of coordination between the six gp16 subunits.^32^^,^^41^^,^^86^ The linker between the N-terminal and the C-terminal domains of phi29’s gp16 may also be involved in DNA or pRNA interactions.^36^ In addition, some research has shown that multiple gp16 hexamers are required for DNA packaging, leading to the hypothesis that gp16 forms an open spiral ring, rather than a closed ring^27^^,^^28^^,^^87^ (Figures 5C and 5D).

### Energy landscape of the sequential action model

Part of the motive forces for ATPase-driven motion is the conformational change of the subunit of the ATPase ring by alternative closing (contracted) and opening (extended) resulted from ATP binding and hydrolysis, respectively. The closing and opening will lead to a sliding or pushing motion. Sliding or pushing might be triggered by the departure of the hydrolysis products ADP or Pi.^88^^,^^89^

During the conformational transition, the conserved residues in the ATPase sequence might play important roles in force generation. A “power stroke” mechanism for ATPase motors^90^^,^^91^^,^^92^^,^^93^^,^^94^ has been proposed. In the power stroke mechanism, the energy-rich gamma-phosphate bond of the ATPase is hydrolyzed. The high-chemical energy release is converted into physical motion via a power stroke similar to the motion of the engine pistol resulting from gasoline burning.^95^ However, the recent evidence from the investigation of the ATPase motor leads to the following data and parameters contradictory to the power-stroke hypothesis: 1) The DNA movement is caused by the conformational change of the ATPase instead of by ATP, ADP, Pi, or the energy directly.^3^^,^^96^ 2) The timing does not match the power-stroke hypothesis because the motion of dsDNA is much later than the ATP hydrolysis step at the time of ADP or Pi release. That is to say, the conformational change of the ATPase subunit is the mechanism that causes motor motion and dsDNA translocation.^58^

It has been reported that the conformational change is accomplished in two characteristic steps: changing of the hydrogen-bond network around ATP and the dynamic movement of an ATPase domain via sliding of one of the subunits.^97^ Sliding enhances the hydrophobic stabilization because of the exclusion of water molecules from the interface and improved packing in the hydrophobic core. This step contributes to a decrease in free energy, leading to the generation of torque in the ATPase subunit on ATP binding.

ATP binding to and hydrolysis by the Walker A and Walker B domains has been well-investigated (Figure 1).^3^^,^^98^ The arginine finger in the ATP-binding domain is the starter of the ATPase motor and the catalyst for the dimerization of the two ATPase subunits.^40^ The above theory that ATP-ATPase binding drives conformational changes in the structure of the ATPase can apply to the binding of phi29’s gp16 complex to dsDNA. ATP hydrolysis drives the gp16 subunit to release the dsDNA, and the subunit follows a conformational path back to its previous state and repeat the cycle after the dsDNA has been passed to the adjacent gp16 subunits.^28^^,^^35^^,^^40^^,^^99^ However, a pressing question remains: Why does dsDNA move to the adjacent downstream ATPase gp16 subunit rather than go back to the upstream subunit of the ATPase hexameric ring? It should be capable of moving to any of the surrounding subunits because they are all relatively equally dispersed. An explanation for this question is that the ATPase subunit has a structural conformation that only allows the dsDNA to move in one direction around the ring where the DNA chirality helps control the direction of motion.^99^^,^^100^ In addition, the motor channel is only 3.6 nm.^23^ The limited space in the channel prevents the dsDNA from having the freedom to move to another subunit instead of the adjacent one.

The 5’/3′ double-stranded end-region, especially the CCA bulge, of the pRNA in the phi29 packaging motor interacts with gp16.^42^^,^^66^^,^^99^ Because of the relative elasticity and dynamic properties RNA,^36^^,^^101^ a possible model for pRNA function as rigid body arms and hinges has been proposed, linking its mechanism to the studies of motion in other RNA systems.^102^^,^^103^^,^^104^^,^^105^^,^^106^^,^^107^^,^^108^^,^^109^ ATP hydrolysis by the motor complex, including gp16 and pRNA, causes relative movements among the three arms extending from the 3-way junction in the RNA monomer, and leads it a contracted state.^110^ Such conformational changes between ATPases, RNA, and ATP have also been observed in DEAD-box helicases.^111^ The sequential action of the gp16-pRNA complex for dsDNA translocation might be caused by this phenomenon.^36^ The role of RNA dynamics in functional regulation and complexity has been seen in most facets of both coding and noncoding RNA systems.^112^ It is apparent that RNA’s “breathing”, kinetics, and elastic properties contribute to its function in the packaging complex.

### Electrostatic interaction of four electropositive lysine layers with the electronegative DNA backbone facilitates advancement along one strand and supports the revolution mechanism

**Figure 6:**
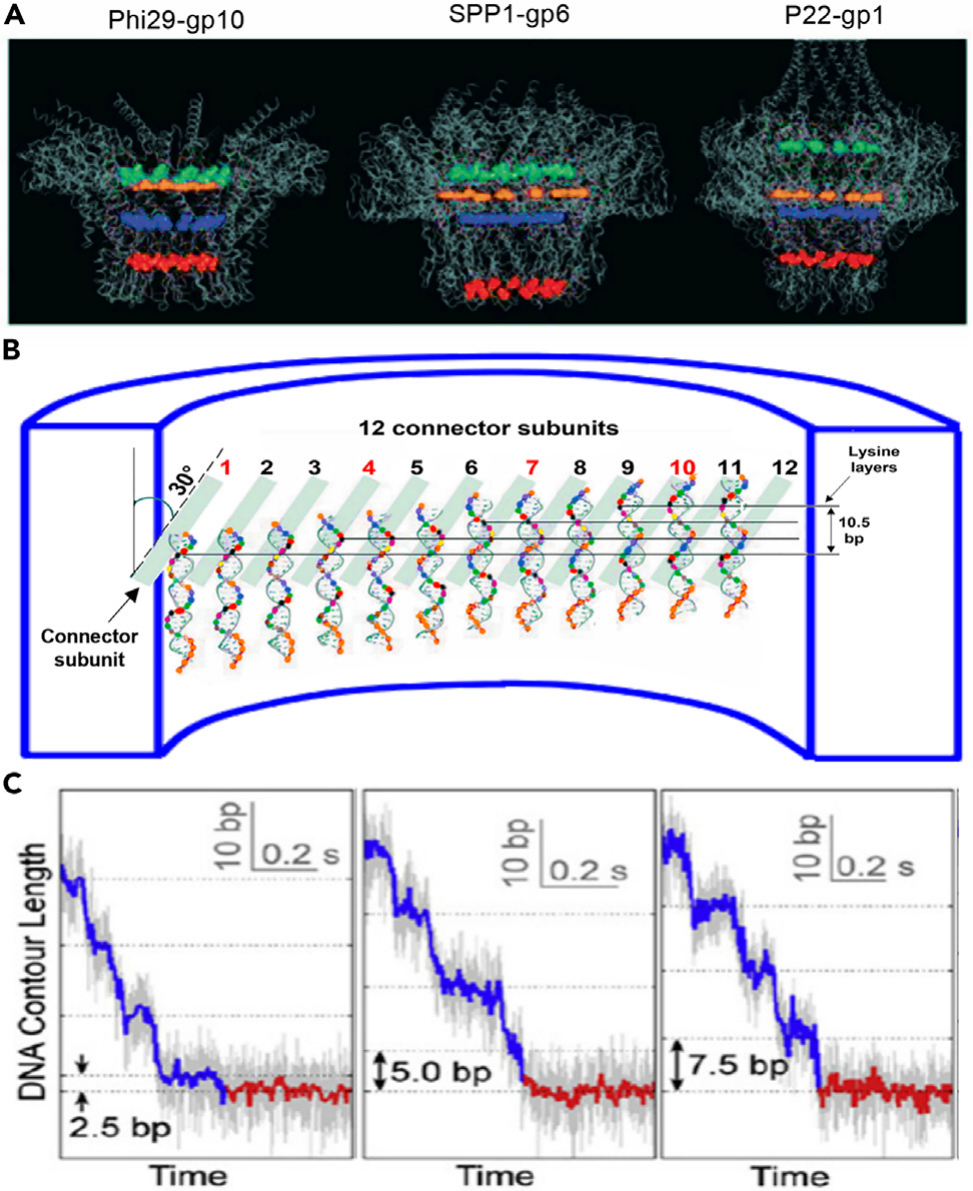
Four Lysine Rings in Bacteriophage Channels (A) Phi29 (PDB:1H5W),^14^ SPP1 (2JES),^113^ and P22 (3LJ5)^114^ each possess 4 rings of lysine which contact one strand of the dsDNA.^27^^,^^77^ (B) Schematic of the 12 connector subunits showing the 4 electrical attractions between the positively charged lysine and the negatively charged phosphate backbone based on uneven and mispaired electrostatic interactions across the channel complex, resulting in four pauses.^27^^,^^33^ The connector has 30° left-handed chirality, which appears right-handed when cut open (see Figure 10). (C) Four rings and four pauses per 10.5 bp will result in about 2.5 bp per pause (or more because of an uneven distance between lysines) with four pauses per turn.^32^

DsDNA translocation motors are elegant, meticulous machines. Several intricate structural characteristics are critical concerning the geometric features of helical dsDNA. These translocation motors all display a multiple-subunit, ring-shaped structure. A common feature of these motors is that the interior surface is mostly electronegative. Such electronegativity repulses negatively charged DNA, preventing DNA from sticking during translocation.^45^ A single lysine within each protein subunit results in the formation of a horizontal ring within the multi-subunit connector. Connector crystal analysis revealed that many phage connectors, such as those in phi29,^14^ SPP1,^113^ and P22,^114^ contain four positively charged lysine layers, which interact with the electronegative phosphate backbone of DNA^24^^,^^27^^,^^77^ (Figure 6A). In the phi29 connector, these four layers are made up of 48 lysine residues distributed among the 12 gp10 subunits.^14^^,^^33^ Each layer is composed of 12 lysine molecules, with each lysine coming from one subunit of the dodecameric connector.

Recent investigations into the detailed interaction of lysine residues with the bacteriophage genome during translocation revealed that the channel wall layers generate force that alters the speed of DNA translocation, resulting in four steps of pausing during DNA advancement.^33^ DNA revolves through each of the 12 connector subunits per cycle, with only one strand touching the channel wall^27^^,^^33^ (Figure 6B). During this revolution, the negatively charged phosphate backbone of dsDNA contacts the positively charged layer of the lysine ring.^28^^,^^33^^,^^115^ The positive-negative charge interaction generates a transient sticking force. This results in an uneven speed, causing the four pauses during DNA translocation^27^^,^^28^^,^^32^^,^^33^ (Figure 6C). Such transient holding will be disrupted by the pushing force of the ATPase.

The role of the positively charged lysine is to hold the negatively charged dsDNA, preventing the DNA from reverse sliding. However, too many positive charges in the channel wall would lead to DNA sticking to the wall, interfering with its forward translocation speed. Thus, the lysine layers provide an interrupting force to hold the dsDNA. The inconsistent number of 10.5 base pairs per revolution and 12 subunits per 360° leads to a 12.5% mismatch between the DNA and the channel protein subunits (1-(10.5/12))∗100 = 12.5%).^33^ This mismatch is compensated when the contact point between the phosphate and the lysine shifts to a spot on the next lysine ring, causing a slight pause in DNA advancement. Nevertheless, the four positively charged layers are seemingly nonessential for motor DNA-packaging activity, because mutation of one of the four lysine rings does not significantly affect DNA translocation.^77^ This indicates that the interaction of lysine and phosphate is only an ancillary force in DNA translocation, not the main force. Such pauses during DNA packaging have been reported in both phi29^32^^,^^86^ and T4.^116^

### Mechanism to prevent DNA from sliding out by three-step channel gating

In the viral replication process, the viral DNA is translocated into the capsid. Inversely, the DNA exits the viral capsid and enters the host cell during the infection process. It was found that the regulation of the DNA translocation was controlled by the following mechanisms: (1) The presence of the one-way valve to control the direction by different loops, (2) The gating of the motor channel to close the channel to block dsDNA sliding and reversal, and (3) The use of the aperture-like motor channel to regulate channel closing after packaging and to facilitate DNA exit during infection.

**Figure 7:**
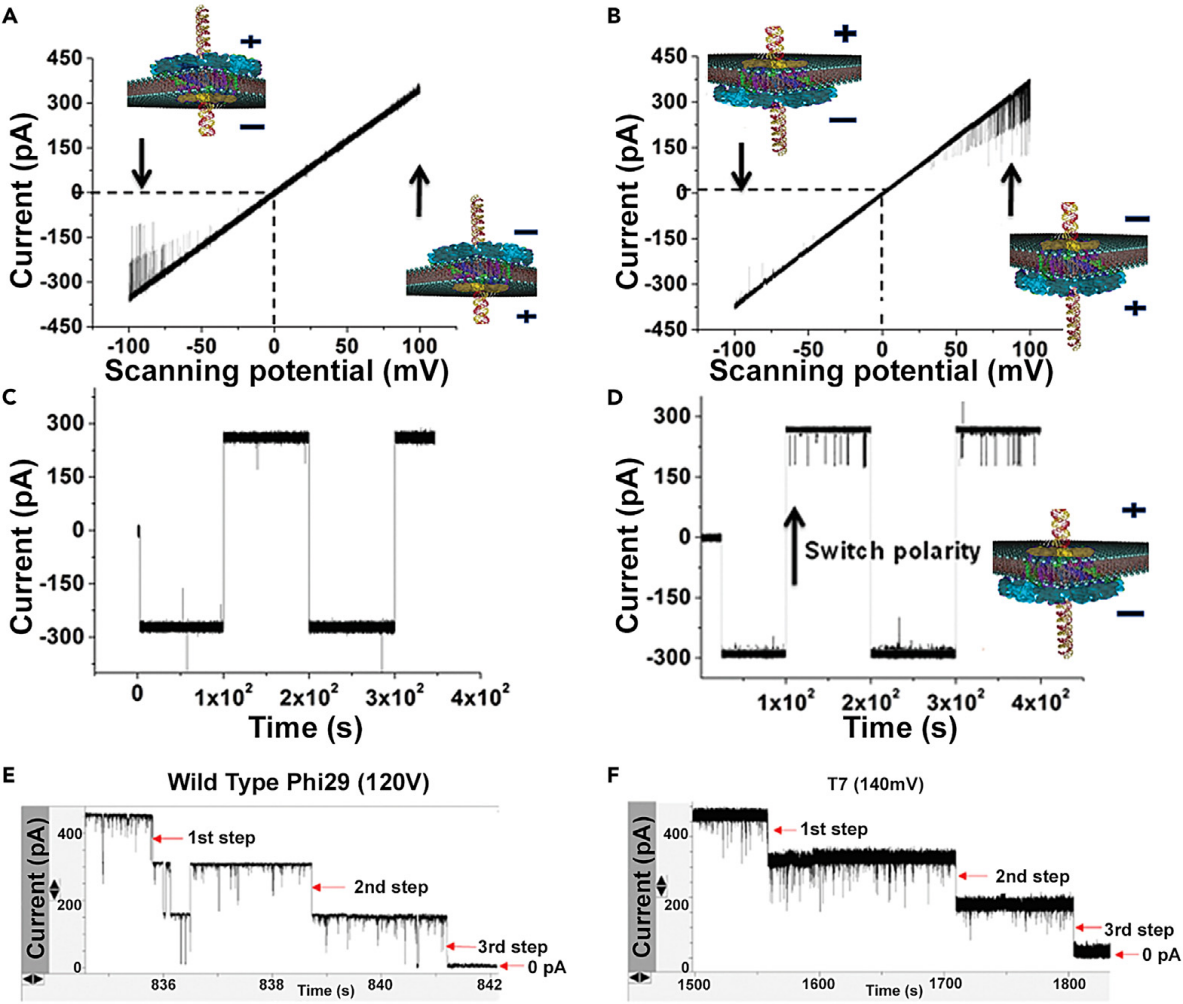
One-way traffic of the phi29 dsDNA packaging motor prevents dsDNA from sliding backward, as evidenced by nanopore translocation studies (A–D) “One-way” translocation of dsDNA through the pore as evidenced by current blockage spikes by ramping voltage (A-B) and switching polarity (C-D). (A-B) The potential at which DNA translocation occurs switches depending on the pore’s orientation. (C-D) DNA translocation can be turned on and off with varying polarity.^29^^,^^33^^,^^65^ (E and F) Three-step gating evidenced by conformational change in the channel pore diameter seen in phi29 and T7 nanopores, respectively.^29^

Phage dsDNA traffics through the connector from the narrower N-terminal to the wider C-terminal through a “one-way valve” mechanism.^65^ In one study, the phi29 or T7 connector was inserted into a planar lipid bilayer membrane.^117^ When dsDNA was added to both chambers, trafficking through the connector was only seen in one direction^29^^,^^33^^,^^65^ (Figures 7A–7D). When the dsDNA enters the procapsid and touches the C-terminal sequence, the interaction results in a 3-step gating that finally leads to the channel gate closing. The channel closes 32% with each step, analogous to a camera’s f-number, as determined by electrophysiological measurements.^118^ The measured potential difference mimics the one that occurs as negatively charged dsDNA is packaged. The conformational change of the aperture-like connector will facilitate the shift in the direction of the one-way valve for dsDNA to exit during the infection process. The 3-step gating process is not exclusive to phi29 and T7 (Figures 7E and 7F), as it has also been documented in T3, T4, and SPP1 connectors.^119^ This is comparable to the conformational changes that occur in the P22 bacteriophage’s DNA channel, called the portal protein.^120^ After the completion of DNA packaging, this portal protein undergoes a major conformational change that may prepare the dsDNA for injection upon infection. The finding of the C-terminal touching for gating and the 3-step gating process has been applied to single-pore sensing for disease diagnosis at a single-molecule level sensitivity.

The discovery that bacteriophage connectors undergo a 3-step gating process during DNA translocation has led to the hypothesis that gp10’s flexible loops play a major role in phi29 gating because, when these loops were removed, the pores exhibited diminished gating.^118^ In the case of SPP1, which also has connector loops, it has been hypothesized that the loops play a role in force-induced conformational changes near the end of DNA translocation.^113^^,^^121^ Further research is needed to validate these hypotheses.

### Asymmetrical structure of the ATPase’s hexameric rings supports sequential action as a mechanism for dsDNA translocation

**Figure 8:**
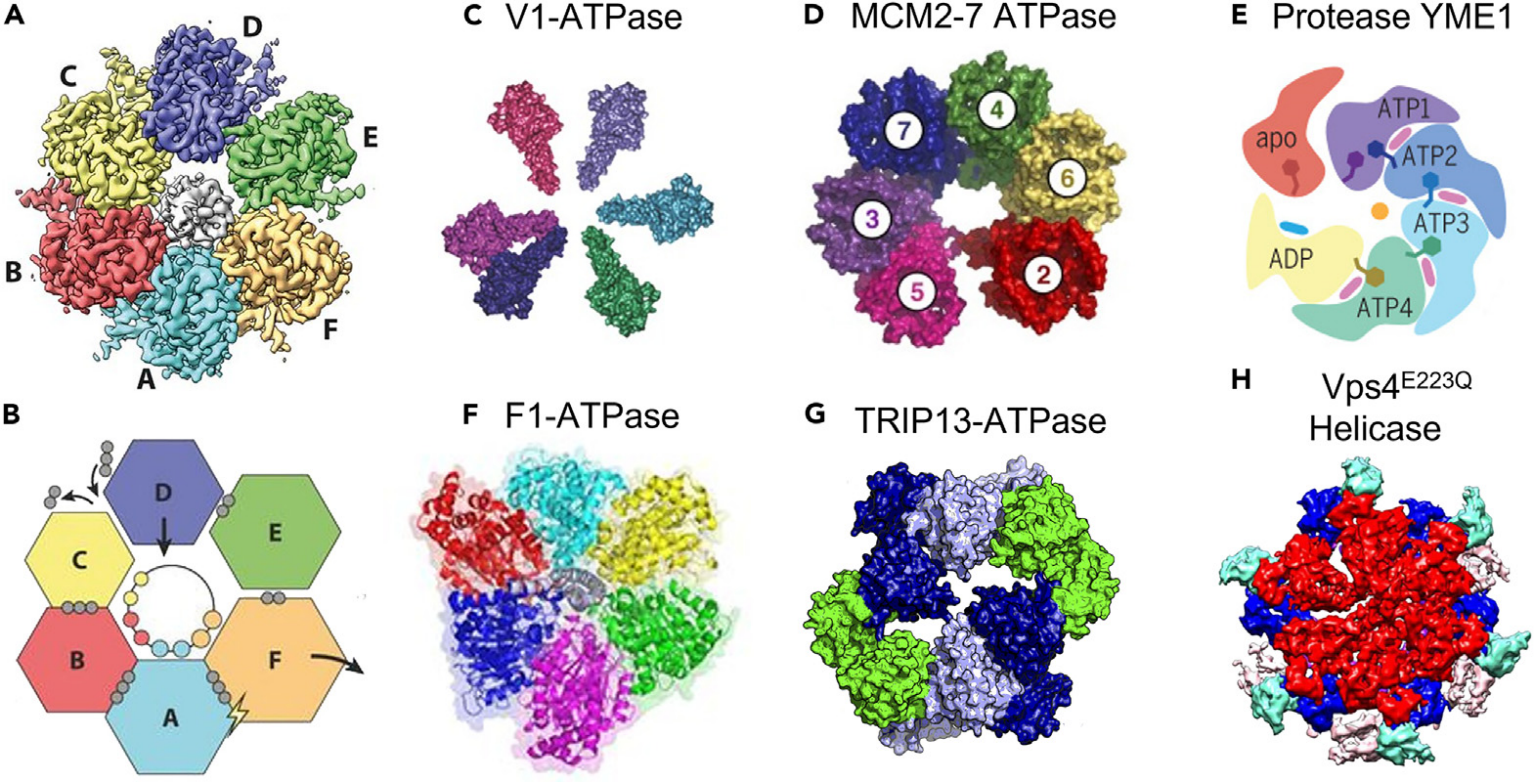
Asymmetry in hexameric biomotors (A and B) CryoEM map and cartoon of asymmetrical FtsK translocating dsDNA, respectively.^43^ (C) V1-ATPase.^129^^,^^130^ (D) MCM2-7 ATPase (reproduced with permission from Wiley).^123^ (E) YME1 protease.^124^ (F) F1-ATPase.^34^^,^^125^ (G) TRIP13-ATPase.^126^ (H) Vps4^E223Q^ helicase.^127^

The tendency of phi29’s gp16 to non-covalently self-assemble and aggregate has been observed via biochemical analysis.^99^^,^^122^ Dimerization occurs when the arginine finger acts as a bridge between adjacent ATPase subunits.^34^^,^^40^ This self-assembly may result in asymmetrical hexameric ring (one dimer and four monomers). Asymmetrical, hexameric ATPase structures have been reported in several other systems including Ftsk,^43^ minichromosome maintenance (MCM) ATPase,^123^ YME1 protease,^124^ F1-ATPase,^34^^,^^125^ TRIP13-ATPase,^126^ and Vps4^E223Q^ helicase^127^ (Figure 8). It is possible that cryo-EM measurements of the phi29 hexameric ring may appear pentamer-like if the resting dimer is interpreted as a monomer (see difficulties in determining the structures and stoichiometries of viral DNA-translocation motors).^128^ Mutation of this arginine finger in gp16 resulted in a loss of self-aggregation, dimerization, and thus packaging assembly consistent with the sequential action model.^34^ This loss of dimerization was rescued when mutant subunits were mixed with subunits containing an intact arginine finger. Nevertheless, a DNA-packaging complex with a single mutated arginine finger still cannot package DNA. Although an intact gp16 can use its arginine finger to dimerize with the mutant, the mutant cannot bind to the next subunit in the sequential process, obstructing packaging.^40^

### Channel pore size permits trafficking of revolving dsDNA

**Figure 9:**
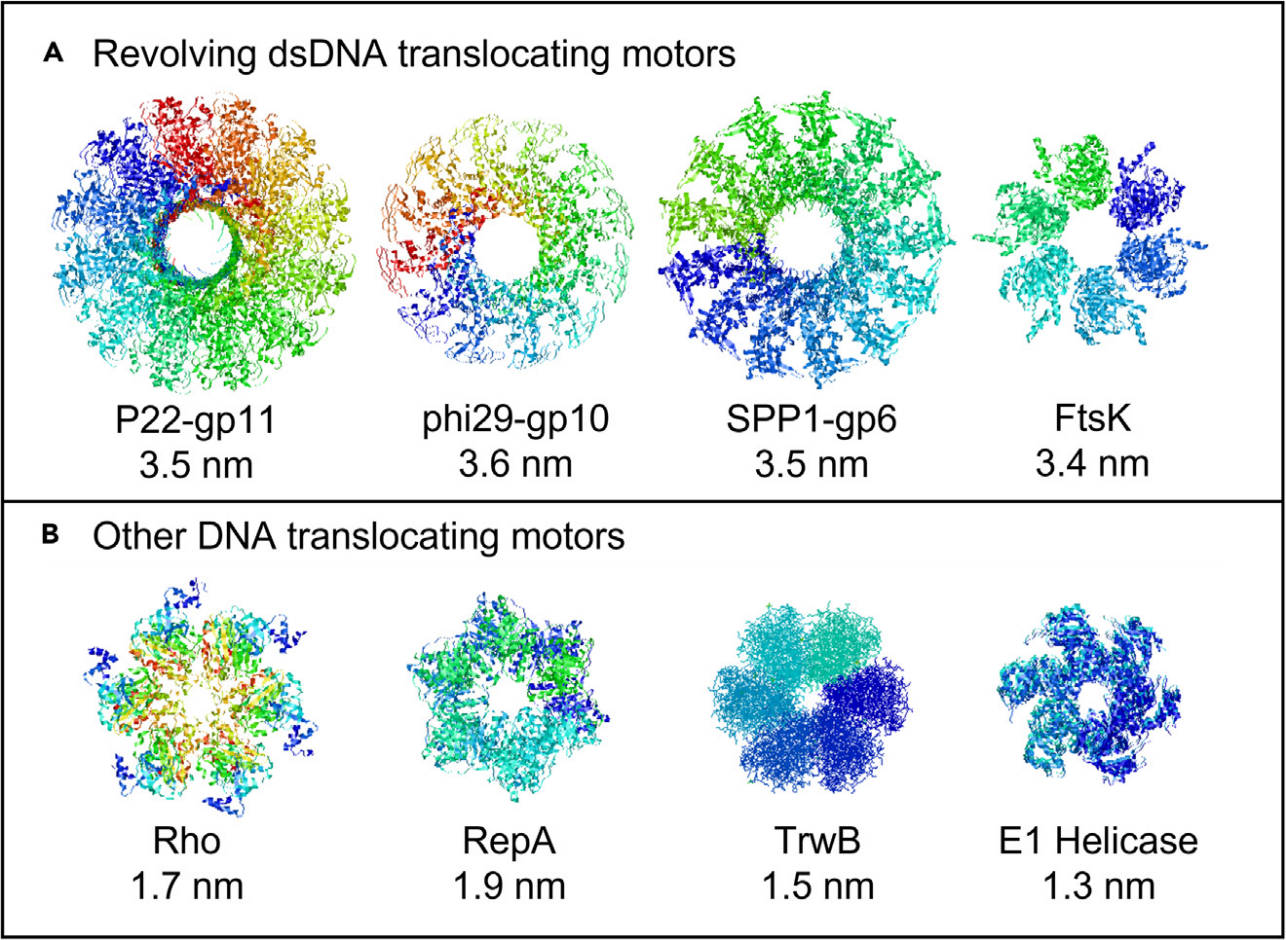
Classification of channel size in dsDNA-translocating motors (A) Revolving motors have pore diameters larger than dsDNA (∼2 nm). Examples include P22-gp1 (PDB:3LJ5),^114^ phi29-gp10 (1FOU),^13^ SPP1-gp6 (2JES),^113^ and FtsK (2IUU).^134^ (B) Other motors have smaller pore diameters. Examples include Rho (3ICE),^135^ RepA (1G8Y),^136^ TrwB (1E9R),^137^ and E1 helicase (2V9P).^138^

In a rotary motor complex, the channel size must be small enough to contact the phosphate backbone of both strands throughout its helical turn during translocation. This puts the hypothesized channel pore size around 2 nm for dsDNA and possibly smaller for ssDNA. However, because of the presence of stacked bases, the diameter of ssDNA is likely larger than 1 nm (50% of 2 nm dsDNA).^131^ A 2 nm or smaller channel size is not seen in revolving of motors such as phi29,^10^ SPP1,^132^ P22,^133^ and Ftsk,^134^ which sit at around 3–5 nm in diameter^24^ (Figure 9).^135^^,^^136^^,^^137^^,^^138^ In revolution channel pores, the DNA backbone contacts the pore complex around the interior edge. This larger channel size is necessary to allow room for revolution because only one strand contacts the channel wall during translocation.^33^ As dsDNA translocates, it must also tilt from the central axis imposed by the motor complex in close proximity to the pore edge.^35^

Meanwhile, the nut and bolt mechanism of action required for rotating DNA, if present, necessitates a much smaller pore size than the diameters observed by X-ray crystallography for the dsDNA translocation motor (Figure 9A). When the channel diameter is significantly larger than that of dsDNA, the nut and bolt “threading” mechanism cannot work. Moreover, if a channel is similar in diameter to dsDNA, it cannot accommodate the tilting that occurs during revolving translocation. CryoEM evidence has uncovered that for the T7 phage, dsDNA must tilt during translocation, which is consistent with the revolving mechanism.^46^

### The 30° chirality of the connector channel supports the revolving motion mechanism

**Figure 10:**
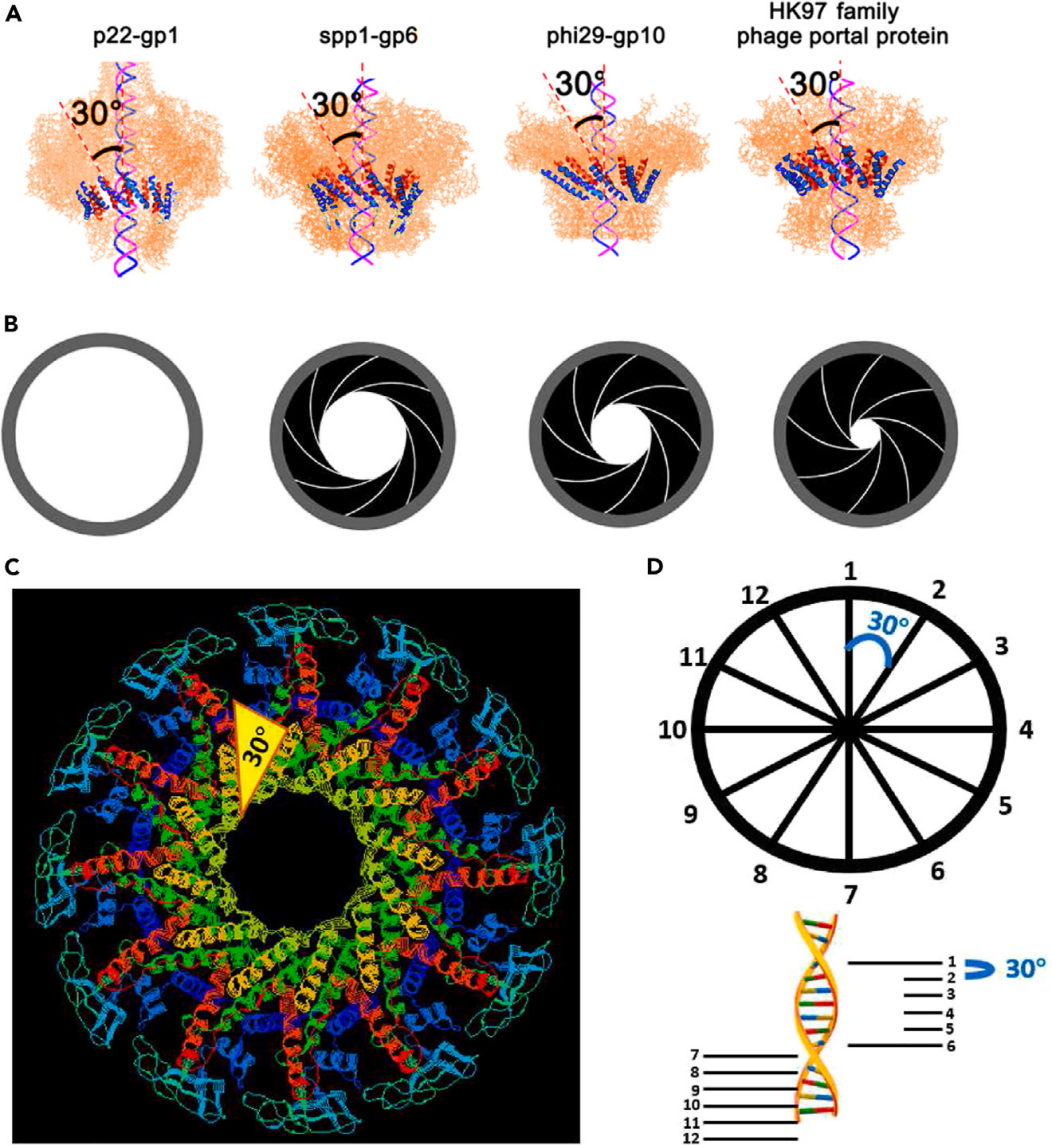
The 30° chirality and mechanism of viral DNA-packaging motors (A) Quaternary structures showing the presence of the left-handed 30° tilting of the connector subunits of bacteriophages: P22 (PDB: 3LJ5),^114^ SPP1 (2JES),^113^ phi29 (1H5W),^14^ and HK97 (3KDR), causing an anti-chiral configuration between the left-handed connector subunits and the right-handed dsDNA helices. (B) 3-step gating is analogous to a camera’s f-stop (aperture). (C) Crystal structure of the phi29 connector revealing the aperture-like structure with 12 gp10 subunits which tilt 30°. (D) A 360° circle divided into 12 sections showing 30° between each section and one 360° turn of DNA divided into 12 steps.^29^

Phi29’s tail connects to the viral capsid by a connector made of 12 gp10 protein subunits arranged in a funnel shape^13^^,^^14^ (Figure 10). Each gp10 subunit contains three left-handed helices surrounding the channel. DNA exhibits right-handed chirality; therefore, the connector channel has antiparallel (left-handed) helices compared to dsDNA.^65^ Each of the 12 gp10 subunits is tilted 30°^29^ (Figure 10A). As dsDNA moves from one gp10 subunit to the next, it completes a 360° revolution along one strand around the connector channel in twelve 30° steps (360°/30° = 12 steps).^33^^,^^38^ The arrangement of the gp10 subunits in this tilted fashion ensures that the 5’→3′ DNA strand remains in contact with the wall of the connector channel during translocation. The subunits of the dodecameric connector of the P22,^49^ SPP1,^113^ and Hong Kong 97 phages also tilt 30° with respect to the central channel^24^ (Figure 10), and the connector shape is similar to that of the phi29 connector, despite little sequence homology.^24^^,^^119^ The 30° tilting ensures the engagement of DNA with the channel wall to prevent backward slipping.

### Difficulties in determining the structures and stoichiometries of viral DNA-translocation motors

Much of what is known about these motors discussed above comes from structural and biochemical studies. As technology has improved, more of these structural findings have come from cryoEM research. In fact, recent advances in cryoEM technology have been described as the “resolution revolution”.^139^ This technological leap was spurred by many advances, including direct electron detectors and single particle analysis. Direct electron detectors allow for direct detection of electron signals, circumventing the need to convert these electron signals into photons, and thus resulting in superior detective quantum efficiency.^140^ In single particle analysis, numerous 2D images of the sample are taken and combined to ascertain its 3D structure.^141^ Other new technologies, such as the Volta phase plate, further improve cryoEM resolution by increasing the contrast.^142^

Despite many groundbreaking advances, cryoEM, with other structural techniques, still has limitations. This is particularly true when studying these revolving DNA-packaging motors.^128^^,^^143^ For the best results, cryoEM and other structural techniques should be combined with biochemical studies to provide a complete account of these motors while translocating DNA. Such biochemical studies include native PAGE, electrophoretic mobility shift assays (EMSA), and capillary electrophoresis (CE).^115^ Some of the obstacles to resolving these structures and stoichiometries are described below.

#### The first challenge is the resolution of a complex structure

**Figure 11:**
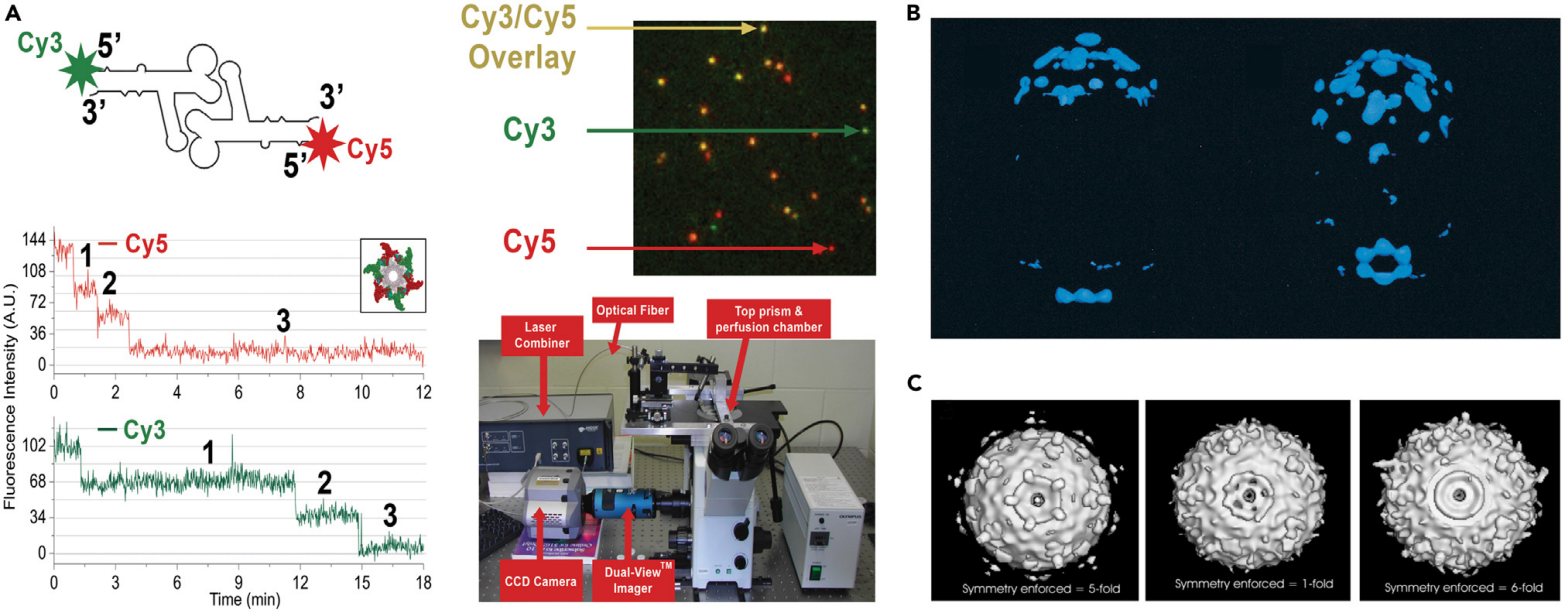
Early cryoEM reconstruction phi29 DNA pndackaging motor (A) Dual-view imaging of procapsids containing both Cy3-a Cy5-tagged pRNA.^15^ (B) From Ibarra et al. 2000, the phi29 packaging motor was observed with a hexameric structure.^143^ (C) CryoEM of the phi29 packaging motor with 5-fold (left), 1-fold (center), and 6-fold (right) enforcement, revealing an asymmetrical structure when a 1-fold was enforced.^128^

One reason why the stoichiometry of most viral ATPases is still debated whereas that of most non-viral ATPases is not is because viral ATPases are generally smaller than cellular ones. Viral DNA-packaging motors are around 7–8 nm, while cellular ATPase complexes are typically larger. Likewise, the molecular weights of phage DNA packaging motors are smaller than those of cellular complexes. For example, phi29’s gp16 is 332 amino acids,^36^ but bacterial FtsK is 1329 amino acids.^144^ Within a complex structural environment, clear resolution of the motor complexes’ detail is challenging. Techniques to increase the size of the motor component might help to address this challenge, but it must be done in a manner that does not interfere with biological activity. In addition, stoichiometry can also be investigated by fluorescent single-molecule studies^15^ (Figure 11A).

#### The second challenge is the difficulty of capturing fast-moving objects with imaging technology

One common feature of viruses is their unusually high and rapid replication rate. The phi29 motor has been found to be the strongest biomotor, as discussed earlier, and it moves very quickly.^145^ Throughout this process, proteins involved in packaging frequently change conformation, making them challenging to measure.^119^ Such difficulty hampers attempts at measuring step size, pause, sliding, and packaging speed using current imaging equipment. Optical tweezers have been a technological revolution and the best technology for measuring motion speed and force.^32^^,^^86^^,^^146^^,^^147^^,^^148^^,^^149^ Regardless, it still has limitations in measuring motion step size and distinguishing the effects of vertical or horizontal factors. Frequent conformational changes also pose challenges for capturing these motors in their intermediate states. Using a blocking agent to freeze the motor in a DNA-packing intermediate might be an approach to address this challenge.

#### The third challenge is the asymmetric structure

The sequential inchworm process can create one dimer and four monomers in a complex, resulting in an asymmetrical structure, such as in phi29.^34^ At any moment, the subunit interacting with the dsDNA would be distinct from the other subunits in the hexameric ring. In the case of phi29’s gp16, it can dimerize even without ATP.^40^ This asymmetry was illustrated by cryoEM images of the phi29 packaging motor which appeared as an asymmetrical structure when 1-fold symmetry was enforced^128^ (Figure 11B, center). However, because of the resolution limit of earlier cryoEM technology, this asymmetrical hexamer was interpreted by the authors as a pentamer because 5-fold enforcement was applied^128^ (Figure 11B, left). Recent advances in single particle construction^150^ and artificial intelligence-based grouping and categorizing have produced high-resolution structures of asymmetric proteins.^151^ Such technology may be applied to bacteriophage motor structure and stoichiometry.

#### The fourth challenge is the presence of a thermodynamically stable 3WJ in pRNA

The isolated pRNA structure has been revealed by both AFM and X-ray crystallography. AFM imaging studies found hexamers^152^; however, crystal structure and single-molecule experiments resulted in hexameric^15^^,^^153^ and pentameric^154^ pRNA structures. Reasons for this discrepancy are discussed below.

The three-way junction (3WJ) of pRNA displays unusually high thermostability and is a critical key in driving pRNA folding.^155^ The 25-nt 3WJ is so stable that it always forms a strong and fixed pseudoknot structure whenever the three individual subunits, 3WJ-a, 3WJ-b, and 3WJ-c, are present, regardless if they are separate or in one parent pRNA sequence.^156^ Based on the thermostability of pRNA 3WJ in driving the formation of a pRNA pseudoknot and the subsequent pRNA ring, the part of its sequence harboring the complete 3WJ has been used to generate crystal structures. In one study, the pRNA crystal structure was assembled into a hexamer,^153^ whereas a different study fitted a pRNA crystal into a pentameric model.^154^ However, it should be noted that the latter study used a pRNA 3WJ core that was substantially truncated and modified. Nucleotides at the 5′AND-3′ ends of the 3WJ were removed, and four nucleotides at the center of the 3WJ sequence were replaced.^154^ The crystal structure from this truncated pRNA generated a tetrameric complex that was used to dock onto the phi29 procapsid, resulting in a computed pRNA pentamer. Although these modifications improved crystal diffraction, they also could have affected the resulting crystal structure because the critical 3WJ was modified. Another explanation for this discrepancy is that pRNA might assemble differently when it is part of the whole motor versus when it is studied separately.

### Applications of viral DNA-packaging motors

**Figure 12:**
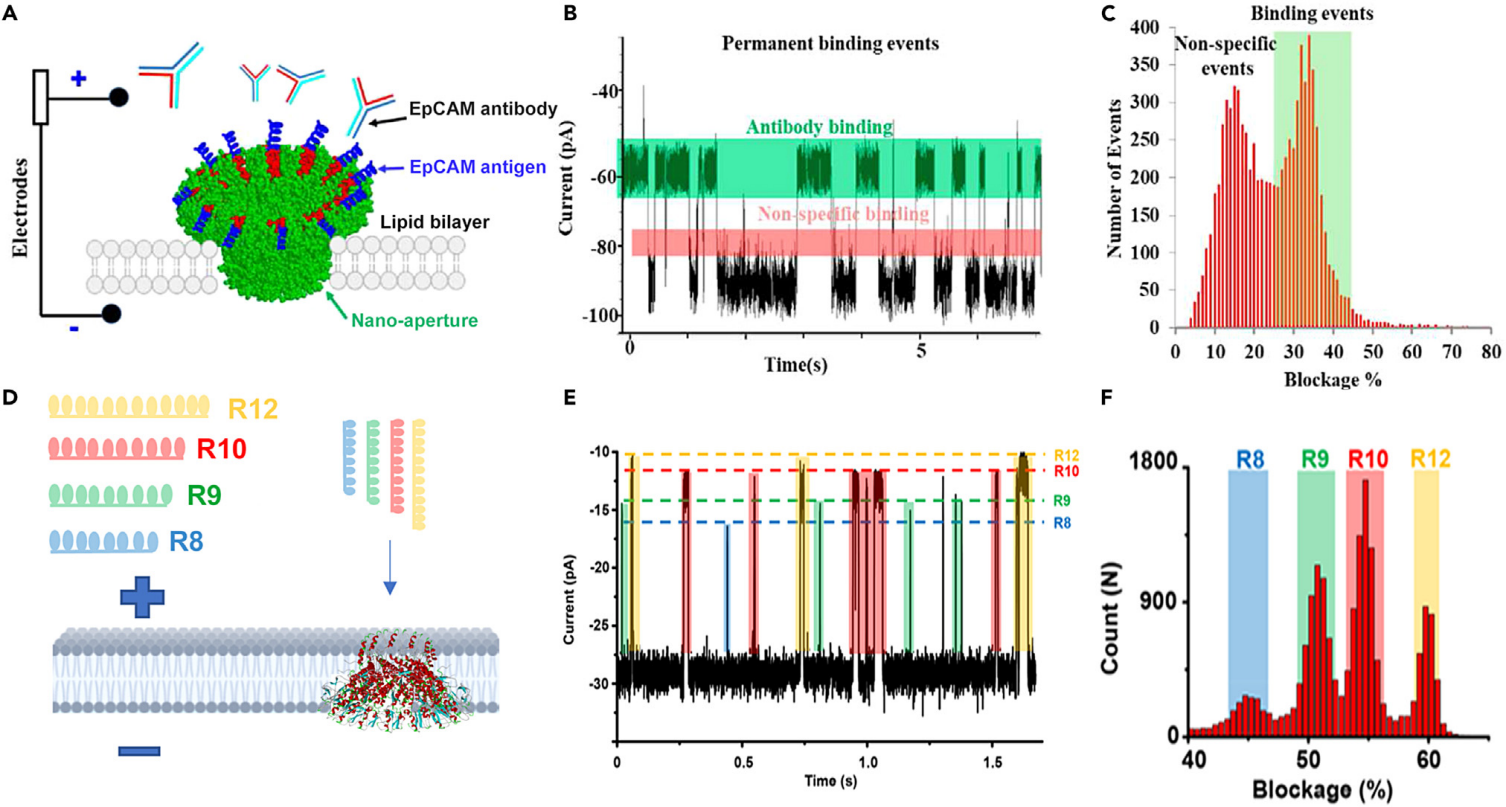
Applications of DNA-packaging motor channels for single-pore sensing and single-molecule diagnostics (A–C) Detection of single antibody via current blockage profiles and dwell times for colon cancer diagnosis.^29^ (D–F) Differentiation of peptides varying in length by a single amino acid (12, 10, 9, and 8 respectively).^158^

Besides the discovery of the motor translocation mechanism as discussed above, decades of fundamental studies on the phi29 DNA packaging motor have led to three major breakthroughs in their medical applications: (1) Studies on the elegant motor channel have led to their application in single-pore sensing of DNA, RNA, and proteins^29^^,^^157^^,^^158^^,^^159^^,^^160^ (Figure 12), (2) Investigations into the hand-in-hand interaction in hexameric pRNA ring formation,^71^^,^^161^ as well as the thermodynamically stable 3WJ, have resulted in the emerging field of RNA nanotechnology for cancer therapy,^155^^,^^162^^,^^163^^,^^164^^,^^165^^,^^166^^,^^167^^,^^168^ and 3) Studies on the motor stoichiometry of homologous multi-subunits have led to the creation of new methods in high-throughput drug development.^169^ As understanding of these motors advances, new applications will be discovered and cultivated.

## Conclusion

The genomes of eukaryotes, bacteria, and many viruses are double-stranded and lengthy. Transportation of dsDNA is a complex process because it requires huge energy consumption to avoid coiling and tangling. During their long evolution, these systems acquired a novel revolving mechanism to translocate and package dsDNA without such coiling and tangling. To accomplish this novel process, the living system has adapted the following structural and functional features: (1) Use of ATP to induce conformational changes of the ATPase motor, (2) An inchworm sequential action mechanism for dsDNA transition, (3) An asymmetrical structure during translocation, (4) A left-handled chirality of the channel wall to ensure forward advancement without retraction, (5) A hexameric structure, (6) A large channel to provide room for dsDNA to revolve, (7) Movement along one dsDNA strand to ensure unidirectional moment, and (8) A negatively charged internal channel with lysine layers, preventing the dsDNA from sticking to the wall and facilitating DNA advancement. This elegant and precise machine evolved for its important function in handling dsDNA in bacteriophages, eukaryotic viruses, bacteria, and possibly other systems.
